# Are There Personality Differences between Rural vs. Urban-Living Individuals of a Specialist Ground Beetle, *Carabus convexus*?

**DOI:** 10.3390/insects12070646

**Published:** 2021-07-15

**Authors:** Tibor Magura, Szabolcs Mizser, Roland Horváth, Dávid D. Nagy, Mária Tóth, Réka Csicsek, Gábor L. Lövei

**Affiliations:** 1 Department of Ecology, Faculty of Science and Technology, University of Debrecen, Egyetem sq. 1, H-4032 Debrecen, Hungary; maguratibor@gmail.com (T.M.); mizserszabolcs@gmail.com (S.M.); horvath.roland@science.unideb.hu (R.H.); nagydavin@gmail.com (D.D.N.); toth.maria@science.unideb.hu (M.T.); csreka994@gmail.com (R.C.); 2 Department of Agroecology, Aarhus University, Flakkebjerg Research Center, DK-4200 Slagelse, Denmark

**Keywords:** carabid, urbanization, exploratory behavior, risk-taking behavior, personality, human disturbance

## Abstract

**Simple Summary:**

Urbanization causes substantial changes in environmental and habitat conditions. These, as well as more frequent disturbance events accompanying urbanization constitute selective forces acting on various reactions of urban-associated species, including behavior. In this study, rural and urban individuals of a forest specialist ground beetle, *Carabus convexus* were tested for their exploratory and risk-taking behavior. Beetles responded consistently in the different contexts, and also over time, demonstrating that they had personalities. *Carabus convexus* is the second ground beetle species in which the existence of personality was demonstrated. By agglomerative cluster analysis, we identified two groups of behavioral traits: the exploratory and the risk-taking dimensions of personality. Urban females were significantly more exploratory than urban males which can be an adaptation to find high quality food needed to mature eggs, as well as to find favorable microsites for oviposition. Moreover, urban females and males showed higher level of risk-taking behavior than rural females. Urban beetles with higher risk-taking behavior may be better able to cope with new conditions created by frequent urbanization-driven disturbance events.

**Abstract:**

The world-wide, rapid urbanization is leading to substantial changes in environmental and habitat conditions. These changes, as well as disturbances accompanying urbanization have considerable effects at various levels of the biological organization on wildlife. Understanding behavioral responses to such changes is essential for identifying which organisms may successfully adapt to the altered conditions. In this study, individuals of a forest specialist ground beetle, *Carabus convexus*, from rural and urban forest patches were tested for their exploratory and risk-taking behavior. Beetles responded consistently in the different contexts; furthermore, by behaving consistently over time, demonstrated that they had personalities. Agglomerative cluster analysis identified two groups of behavioral traits: the exploratory and the risk-taking dimension of personality. Urban females were significantly more exploratory than urban males which can be an adaptation to find high quality food needed to mature eggs in urban habitats, as well as to select favorable microsites for oviposition. Moreover, urban females and males showed more risk-taking behavior than rural females. Urban beetles with more risk-taking behavior may be better able to cope with frequent urbanization-driven disturbance events.

## 1. Introduction

Urbanization is a fast increasing component of global change. It is a process during which more area is under urban land use, accompanied by the growth of urban population and the spread of urban life-form [1]. Urbanization can destroy, modify and/or fragment natural habitats [2]. Fragmented natural habitat patches in urban areas often become isolated, limiting species dispersal and nutrient flows between patches [3,4]. Urbanization also profoundly modifies pollutant deposition [5], various climatic parameters [6,7], amounts of nutrients [8], and several biological processes, such as decomposition and mineralization [9], gene flow [4], and community assembly [10].

Modifications in the structure, composition, and environmental parameters of urban habitats generate pressures on organisms living there, (often adversely) modifying their activity pattern, spatial distribution, phenology, body condition, productivity, behavior and biotic interactions [1,11,12], with can subsequently trigger changes in the structure and composition of urban biotic communities [13,14,15], including those of mammals [16,17], birds [18,19], or plants [20,21]. Terrestrial arthropods are less studied (but see [22]); but existing data indicate that urbanization is a global threat to insect diversity [23]. Of the terrestrial arthropods, ground beetles (Coleoptera: Carabidae) have been a favorite group for urbanization studies [24,25,26] for reasons of their diversity, abundance and the availability of an appropriate methodological toolkit [27], and considerable effects at various levels of biological organization are well documented [11].

Organisms can and do react to changes in their environment at various levels. Changes in behavior is perhaps the fastest way of reacting, and such reactions are very predictable in response to certain unfavorable changes in environmental conditions. However, behavior is much more complex than a set of reflective reactions to triggers, and it was found that individual animals can react consistently to environmental conditions experienced through their lifetimes, i.e., have personalities [28,29]. To what degree can such personality traits be interpreted as adaptive plasticity are currently little understood.

Urban environments create distinct environmental conditions and pressures that select for specific traits [13,30]. For example, species with wide tolerance limits (habitat generalists [31]) or with traits that increase tolerance to urbanization-generated conditions [32] are clearly beneficial. Some behavioral traits, such as high exploration and high risk-taking, can be beneficial to cope with urban environmental conditions and/or for colonizing these habitats [11,33]. The only urban study on behavioral traits of ground beetles [33], however, raised questions about the consistency of individual behavior. Different species and even the same species in different localities could show different responses to urbanization, preventing to draw generalizations for ground beetles. At the species level, specialists are more sensitive to changes accompanying urbanization than non-specialist ones [15,24]. To date, however, behavior in differently urbanized habitats of only one habitat specialist ground beetle was tested, that of the harpaline *Pterostichus oblongopunctatus* (Fabricius, 1787) [33]. Previous studies on vertebrates provide evidence that urban individuals are more exploratory and bolder than their rural conspecifics [34,35]. Therefore, we hypothesized that ground beetles in urbanized habitats should also be bolder and display more exploratory behavior than their conspecifics living in rural habitats.

In the present study, we found consistent personality differences in exploratory and risk-taking behavior in the forest specialist ground beetle *Carabus convexus* Fabricius, 1775. Urban females were significantly more exploratory than urban males, and urban individuals of either sex showed more risk-taking behavior than rural females. The female-male differences can be explained by resource exploitation behavior, because mature females are actively looking for food to produce eggs, and for suitable microsites for egg-laying.

## 2. Materials and Methods

### 2.1. Study Area and Sampling Design

We collected beetles from four rural forest stands of an extensive forest, near the lowland city of Debrecen (47°32′ N; 21°38′ E) in eastern Hungary, as well as from four urban forest fragments in the city itself. All eight sites were at least 3 ha and at least 250 m from each other (mean distance between the rural sites: 396.5 m; between urban sites: 702.2 m). All selected forest stands/patches belong to a once-continuous old forest (>130 years) dominated by English oak (*Quercus robur*). The current area of the forest is 1082 ha, and despite its contraction as the city grew, still large enough to allow numerous forest-associated plant and animal species to maintain self-supporting populations [36]. The level of urbanization was characterized by the relative built-up area in a 1000 m radius circle around the selected areas, measured by a GIS program using aerial photographs. In the rural, continuous forest there was no built-up area, while in the urban area, >60% of the surface was built-up or drastically different from the original, forested habitat. In the rural forest stands, there was no regular forestry intervention, and the presence of people was minimal, while in the urban fragments, the larger fallen branches and trunks were cut up and left on the ground, and the shrub layer was strongly thinned. Paths were asphalt- or gravel-covered, and human disturbance was considerable.

Ground beetles were collected during the spring breeding period, between 6 April–28 May 2020 using 15 live, unbaited pitfall traps at each site, (2 areas × 4 sites × 15 traps = 120 traps in total). Traps were installed in a random arrangement, at least 10 m apart from each other, and at least 50 m from the nearest forest edge in order to avoid edge effects [37]. Traps were plastic containers (170 mm long × 110 mm wide × 105 mm deep) with shredded leaves to allow small arthropods to hide to prevent predation by larger ones. Traps were covered with a 20 cm × 20 cm piece of fiberboard to protect them from litter and rain. Traps were controlled twice weekly. Trapped beetles were transported to the laboratory, identified to species level, sexed, and settled individually in Petri dishes with moist filter paper (diameter: 90 mm).

### 2.2. Test Organism

The study species, *C. convexus* is a very widespread Eurasian species, with mainly nocturnal activity [38]. In Central-Europe, this predatory species reproduces in early spring and becomes active during the first half of April. In general, oviposition takes place from the middle of April onwards [38]. Teneral individuals appear from the end of July and young adults go to overwinter in November. This wingless (brachypterous), moderately large (14–23 mm) species has limited dispersal power [38]. In the studied region (Great Hungarian Plain) *C. convexus* is a forest specialist species [39]. In previous studies *C. convexus* was categorized as a very sensitive species to urbanization-driven changes [25,40]. In the studied location, its occurrence in urban forest fragments is sporadic and its abundance is also significantly lower than in rural forest stands [41]. The above mentioned characteristics (habitat specificity, large size, limited dispersal power) suggest that *C. convexus* may be a potential candidate as an indicator species of the effects of urbanization [11].

### 2.3. Testing, Evaluating and Measuring Behavioral Parameters

In the laboratory with standardized conditions (natural L:D cycle, 24 °C temperature, 40% RH), individual beetles were left to rest for 2 h with access to water but no food. After this resting period, beetles were individually tested. First, we measured their activity in a novel environment, also referred to as “open field” test [42,43], which is often applied to assess exploratory behavior [33,44,45]. The novel environment consisted of an open white plastic box (364 × 230 mm), on the bottom of which 35 equal-sized squares were marked. At the beginning of the test, the individual was placed in the central square and was covered with a Petri dish (diameter: 55 mm) to calm it down. As soon as the beetle stopped moving around, the lid was lifted, and the behavior was recorded for 90 s using a GoPro HERO6 camera (CHDHX-601-FW). Videos were analyzed using the *pathtrackr* package [46]. We registered the number of squares crossed by the individual (squares visit hereafter), the covered distance (in mm), and the total amount of time spent in motion (sec; motion time hereafter). We also recorded the number of squares not adjacent to the wall that were crossed (inner square visit hereafter), and the time when the individual reached the wall of the plastic box (wall time hereafter). Square visit, covered distance, motion time, and wall time are commonly used as a measure of activity level and exploration in invertebrates [33,45,47,48], while the inner square visit is considered a parameter of both exploration and risk-taking/boldness [45].

Directly after the novel environment test, we tested individuals for their reaction to threats. The fleeing reaction of individuals to a threat was measured using a ring-shaped arena created by gluing a 55 mm-diameter Petri dish in the middle of a larger one (diameter: 90 mm), thus forming a 35 mm wide ring [45]. With lines drawn under the arena and intersecting in the center, the arena was virtually divided into eight even segments [45]. Beetles were individually placed in the arena and allowed to habituate. When they stopped moving, fleeing behavior was provoked by a mechanical stimulus: the beetle was gently hit on its back with a small forceps. The time spent running (flight duration) and the number of segments crossed (flight distance) by the beetle were recorded. The test ended once the insect stopped moving. Flight duration and distance measures are a personality dimension linked to reaction to threat [45]. After every tenth trial run or if the individual has left some excretum, the arena was cleaned with 70% ethanol.

To assess the repeatability of behavior through time, the tests were conducted twice for each individual, with 24 h between the two occasions. This interval is appropriate to assess the repeatability of the measured behavioral variables. Testing individuals over only two trials prevents the habituation to the experimental conditions [45,49].

### 2.4. Statistical Analyses

The effect of urbanization (non-urbanized vs. urbanized) on the behavioral traits was tested with generalized linear mixed models (GLMMs) using the *lme4* package [50]. The probability distribution that best fitted our response variable was checked using the *car* [51] and *MASS* [52] packages. Based on these examinations, we modeled the response variables with count data (square visit, inner square visit, flight distance) using a Poisson distribution with log-link function, while for the other response variables (covered distance, motion time, wall time, flight duration) we used normal error distribution with log-link function [53]. Fixed effects included urbanization level, sex of the tested individual, as well as their interaction. In the models we also considered the nested design of our sampling (sampling sites were nested within the sampling areas). In the models on behavioral measures, trials were regarded as repeated measures, and the person performing the test (observer) was added as random factor. When GLMM revealed a significant difference between the means, the LSD test was used for multiple comparisons among means [53].

To investigate whether the behavior of beetles was consistent across contexts, Kendall’s coefficient of concordance involving all behavioral measures (mean values for the two trials) was calculated using the *DescTools* package [54]. To test the consistency of behavior over time, Spearman rank correlations were computed to assess rank consistency of individuals in their behavioral measures between the trials using the *RVAideMemoire* package [55], as well as repeatabilities from the GLMMs with the individual IDs as a random term were estimated using the *rptR* package [56]. To detect possible associations among the different behavioral measures defining personality dimensions, an agglomerative cluster analysis was made [45,48,57]. A dissimilarity matrix was computed for the behavioural measures (mean values for the two trials) using the Spearman rank correlations (one minus the absolute value of the correlation coefficients). Subsequently, an agglomerative clustering with the Ward fusion method was performed using the *cluster* package [58]. Personality dimensions (clusters of correlated behavioral measures) were identified by examining the average overall silhouette width values for the given number of clusters [59].

## 3. Results

During the reproduction period of *C. convexus* (from April to May) we sampled 37 individuals in the studied habitats. All individuals had sharp or minimally worn mandibles, indicating that they were overwintered individuals in their first breeding season. Twenty-five individuals (15 females and 10 males) were caught in the rural sites, while 12 beetles (7 females and 5 males) in the urban ones.

**Figure 1:**
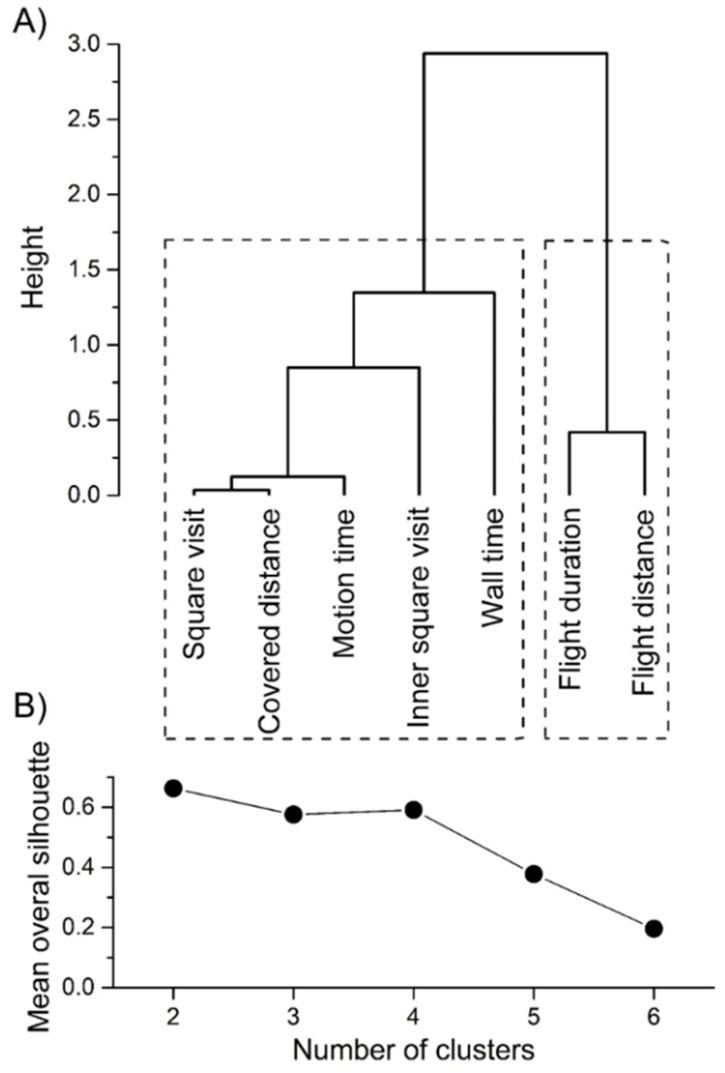
The relationship between the tested behavioral measures of *C. convexus* by agglomerative cluster analysis using the Spearman rank correlations (agglomerative coefficient: 0.8431) and the possible personality dimensions indicated by boxes with dashed lines (**A**), moreover the silhouette plot to identify possible groupings of the variables (**B**).

Significant Kendall’s coefficient of concordance (W = 0.7821, χ^2^ = 173.62, df = 6, *p* < 0.0001) indicated that beetles responded consistently in the different contexts, meaning that beetles were similarly ranked by all behavioral measures. *C. convexus* individuals behaved consistently over time as all behavioral measures tested (square visit, covered distance, motion time, inner square visit, wall time, flight duration and flight distance) were significantly rank-consistent and/or showed significant repeatability between the two successive trials (Table 1). Based on the agglomerative cluster analysis and the assessment of the average overall silhouette width, the studied behavioral measures could be divided into two groups (Figure 1). The first group consisted of square visit, covered distance, motion time, inner square visit, and wall time, and it can be considered to be the exploratory dimension of the beetle personality. The second group included flight duration and distance, and it can be linked to the risk-taking dimension of the personality. Within each group, all behavioral measures were significantly correlated with each other. Between the behavior measures belonging to the two different groups, none of the correlations were significant (Table S1).

**Figure 2:**
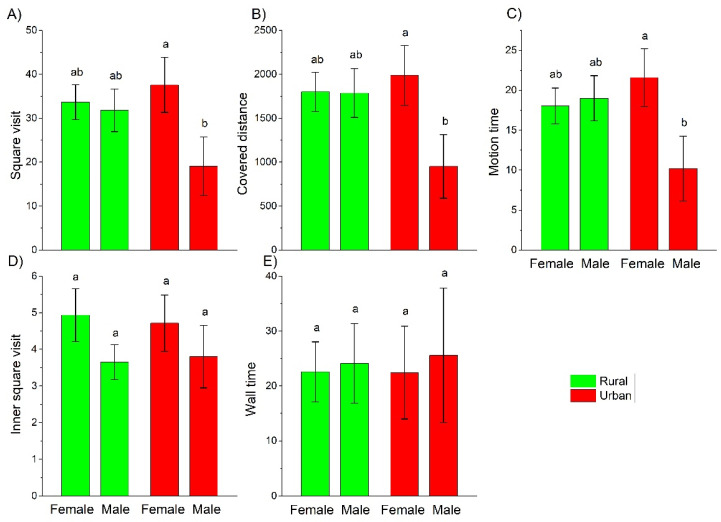
Mean (±SE) values of the tested behavioral measures grouped together in the exploratory dimension of *C. convexus*’ personality by agglomerative cluster analysis: square visits (**A**), covered distance (**B**), motion time (**C**), inner square visits (**D**), and wall time (**E**). Different letters indicate significant differences based on the LSD test (*p* < 0.05).

Three behavioral measures of the exploratory dimension of beetle personality showed similar patterns: square visit, covered distance, and motion time of urban females were significantly higher than those of urban males (Figure 2). In addition, urbanization level and sex were significant factors explaining the covered distance and the motion time in *C. convexus* (Table 2). The other two measures of the exploratory dimension of beetle personality, inner square visit and the wall time were not significantly different among sexes living in habitats with different urbanization levels (Figure 2).

**Figure 3:**
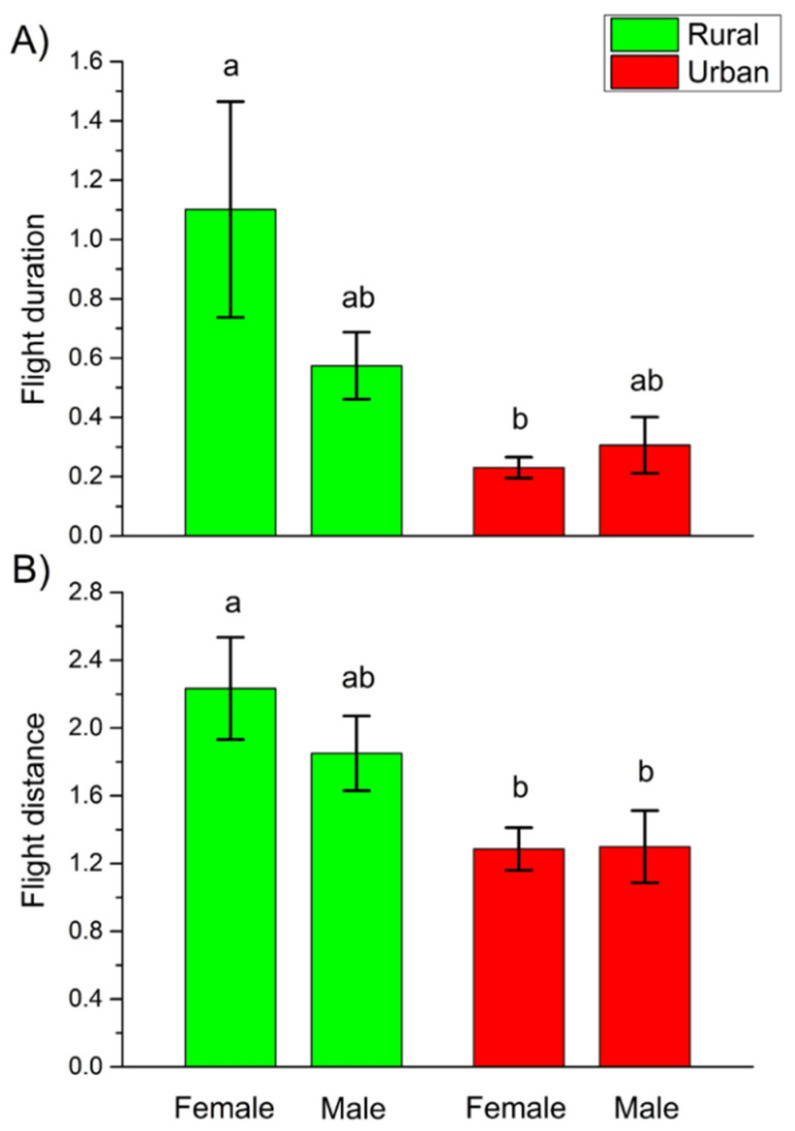
Mean (± SE) values of the tested behavioral measures grouped together in the risk-taking dimension of *C. convexus*’ personality by agglomerative cluster analysis, flight duration (**A**) and flight distance (**B**). Different letters indicate significant differences based on the LSD test (*p* < 0.05).

Regarding the risk-taking personality dimension, there were significant differences in flight reaction between rural and urban females. Urban females showed more risk-taking behavior than rural ones, as the flight duration and distance of urban females were significantly lower than those of rural females (Figure 3). Furthermore, there was a significant difference in the flight reaction between the rural females and urban males, urban males showing significantly shorter flight distance than rural females (Figure 3).

## 4. Discussion

### 4.1. Personality

Our results on seven behavioral measures showed that *C. convexus* individuals behaved consistently over time (between the two trials) and across context (among the different behavioral measures). Thus, we offered evidence for the presence of personality (according to the definition of Sih et al. [60]) in *C. convexus*. To our knowledge, only one previous study described the existence of personality among ground beetles, in *Nebria brevicollis* (F. 1792) [45]. Contrary to several previous behavioral studies working on individuals reared in the laboratory, both Labaude et al.’s [45] and our study were performed on free-living individuals. In the study on *N. brevicollis*, the exact age and the sex of individuals were not determined, thus some of the behavioral variation might be explained by these features [45]. The results obtained in the present study, however, are not likely to have been influenced by these characteristics, since all individuals were of the same cohort (overwintered beetles in their first breeding season) and sexes were distinguished. Although a few previous studies on beetles showed no differences in behavior between sexes [47,61,62], ours and other findings [33] on ground beetles, however, reveal the existence of sex-dependent behavioral differences.

Two personality dimensions or behavioral trait groups could be identified by agglomerative cluster analysis: the exploratory dimension (square visit, covered distance, motion time, inner square visit, and wall time) and the risk-taking dimension (flight duration and flight distance). The number of squares crossed by individuals and motion time (total amount of time an individual was mowing) were also classified as parameters of activity/exploratory behavior of ground beetle individuals [45]. Similarly, covered distance and motion time were grouped together and considered to be related to activity or exploratory behavior in adult leaf beetles (*Phaedon cochleariae* F., 1792) [47,63]; *Galeruca tanaceti* L. (1758) [64]). The categorization of the movement in the central area of the test arena is equivocal, since some studies, including ours, considered it as a parameter linked to activity or exploratory behavior [45,64], while Tremmel & Müller [47] classified this as a measure of boldness. Individuals leaving the safe edge zones to explore the central area of the arena would be more exposed to threats (risk of a predator attack, for example), thus inner zone visit can be associated with both exploration and boldness [45,65]. The time an individual needed to reach the wall of the test arena (wall time) can be considered to be the shyness-boldness dimension of personality in leaf beetles [47,63,64], while an earlier study on firebugs [48] and the present one categorized this parameter as the exploratory axis of arthropod personality. Flight duration and flight distance, parameters relative to threat, were clustered into separate groups in both our and the only previous ground beetle study [45]. Death-feigning reaction, also known as thanatosis or tonic immobility [66] is more frequently used to test beetle boldness or reaction to threats [47,63,64] than flight reaction. There is, however, a trade-off between these two anti-predator strategies as during death-feigning, the individual remains motionless, reducing chances of predation, but remains close to the predator, while during flight the beetle can get away from the predator, but the chances of attack by visually hunting predators increase [67]. Therefore, it would make sense to examine both death-feigning and flight reactions simultaneously. In a previous study, however, only 21% of ground beetle individuals showed thanatosis [33], suggesting that ground beetles are more likely to flee when faced with a threat [45]. Indeed, in preliminary experiments, *C. convexus* individuals turned on their backs continued to move their legs, trying to turn themselves around, and did not show thanatosis (T. Magura, unpublished observation).

### 4.2. Behavioral Differences

In our experiments, urban *C. convexus* covered more distance and spent more time walking than rural ones. Significant urbanization level × sex interaction in square visit, covered distance, and motion time, however, indicated that the exploratory behavior of females and males depended on the urbanization level of their habitats. Indeed, exploratory behavior measured by these parameters on rural females and males did not differ significantly, while urban females were more exploratory than urban males. Females usually invest more into reproduction than males, thus life-history theory predicts marked differences in behavior between sexes [68]. Based on this, as our results also underline, the sex of study objects must be considered during behavioral studies. The only study examining the exploratory behavior of invertebrates in forest patches with different levels of urbanization (low vs. high) within the city of Hamburg found that ground beetles were more mobile in urbanized than in less urbanized sites but only in one of the two-year study [33]. In the year in which the difference was found, only one test was conducted in the field, thus the consistency and repeatability of this behavior could not be evaluated. As the interaction between sex and urbanization level was not tested, and forest patches with low level of urbanization were also within the city, being suburban rather than rural sites [33], our results are unfortunately not clearly comparable with those by Schuett et al. [33]. Nevertheless, studies on birds [69,70] and mammals [35] support that individuals in urban habitats are more exploratory than rural ones.

Our study is, to our knowledge, the first to test risk-taking behavior (or boldness) of beetles from habitats with different urbanization levels. We found significant differences in both the duration and the distance of beetles’ flight behavior between rural and urban females, indicating that urban females were more risk-taking than rural ones. Moreover, urban males also showed significantly lower flight distance than rural females. Beetles consuming low-quality food are bolder than those feeding on high quality food [47]. As urbanization-driven environmental changes usually result in lower food quality [71], urban individuals are expected to be bolder than their rural conspecifics. Indeed, urban mammals [72], birds [70], and reptiles [73] take more risks, while only weak evidence in boldness difference was found for invertebrates [74,75].

Significant difference in exploratory behavior between urban females and males of *C. convexus* can be attributed to the difference in reproductive investments by females and males. As producing and ripening eggs needs extra energy, females have to invest more into reproduction than males. Females take this extra energy from diverse extra food and/or high quality food items [27]. Activity and exploratory behavior is likely constrained by the energy uptake [47]. Indeed, hunger level, defined as the relative gut content is thought to be one of the main driving forces of ground beetle activity [76]. Beside the food limitation under field conditions [77], the gut capacity may also influence the amount of food that can be consumed [76]. Gut capacity is highly influenced by other products stored in the body, such as eggs. Therefore, gut capacity of females maturing eggs is low, so they can only eat small quantities of food at a time, highly influencing their activity and exploratory behavior [76]. In addition, the lower availability, diversity and quality of food in urban habitats [71] may also increase both the distance and the time of the females’ movement needed to find the necessary amount/quality of food [27]. For ground beetles, the dietary advantages of mixed, diverse food over undiversified food items are well known, as is the fact that females consume usually more prey types than males [27]. Furthermore, the existence of fewer potential food items in the studied urban sites was demonstrated by a previous study [39]. Another factor explaining the higher exploratory behavior of urban females, can be the searching for favorable microsites for oviposition. As *C. convexus* individuals do not guard their eggs [38] and egg mortality is significant under field condition [27,78], searching for appropriate egg-laying microsites is essential to ensure egg survival and successful larval hatching [27]. Increased activity and exploratory behavior, however, may enhance predation risk [27]. Consequently, females are forced to compromise between maximizing the likelihood to find appropriate prey items and/or egg-laying microsites and minimizing predation risk [79]. This conflict is less acute in urban habitats where predation pressure is lower [12,80], allowing increased exploratory behavior in these habitats.

More risk-taking found in urban females (and in the case of flight distance, also in males) compared to rural females may be related to disturbance. Differently urbanized habitats differ in the frequency and intensity of anthropogenic disturbances, such as mowing, thinning, vegetation cutting and human attendance but urban habitats are generally more disturbed than rural ones [1]. The behavior of urban dwellers are adapted to these regular disturbance events resulting in more risk-taking behavior during their usual activities, such as searching for food, mating partners, and suitable microsites for resting, reproduction and hibernation [81].

## 5. Conclusions

Our results showed the existence of personalities in *C. convexus*. Urbanization has considerable effects at the behavioral traits: the exploratory behavior of females and males depended on the urbanization level of their habitats. In the rural area, there was no difference in female vs. male exploratory behavior, but urban females were more exploratory than urban males. Increased exploratory behavior of urban females could be explained by the searching activity for high quality food required to mature eggs, as well as by looking for favorable microsites for oviposition. These could suggest the fitness consequences of personality differences. Moreover, urban females and males have more risk-taking behavior than rural females, indicating the adaptation to urbanization-driven disturbance events and threats.

## Figures and Tables

**Table 1:** Summary of consistencies for the behavioral measures of the two trials from Spearman rank-correlation (RS) and (adjusted) repeatability (r). Values in bold denote significant (*p* < 0.05) consistencies.

Behavioral Variable	Spearman Rank-Correlation RS [95% CI] *	Repeatability, r [95% CI] *
Square visit	**0.5201 [0.1571; 0.7516] **	**0.518 [0.140; 0.687]**
Covered distance	**0.4921 [0.1709; 0.7486]**	**0.474 [0.195; 0.686]**
Motion time	**0.4578 [0.1085; 0.7173]**	**0.431 [0.116; 0.668]**
Inner squares visited	**0.4665 [ 0.1597; 0.7094]**	**0.313 [0; 0.518]**
Wall time	0.2382 [−0.1131; 0.5569]	**0.402 [0.088, 0.644]**
Flight duration	**0.5723 [0.2980; 0.7831]**	0.210 [0; 0.513]
Flight distance	**0.3967 [0.0656; 0.6983]**	0.042 [0; 0.188]

* Confidence intervals (CI) was calculated using 1000 bootstraps.

**Table 2:** Summary of GLMM results and post hoc tests on behavioral measures of *C. convexus* in differently urbanized (non-urbanized vs. urbanized) forested habitats (*p*-values in bold denote significant effects).

Response Variable	Fixed Effect	Estimate ± SE	χ^2^	df	*p*
Square visit	Urbanization level	−0.7512 ± 0.4271	3.0938	1	0.0786
	Sex	0.0696 ± 0.3125	0.0497	1	0.8236
	Urbanization level × Sex	1.0114 ± 0.5321	3.9231	1	**0.0476**
Covered distance	Urbanization level	11.4500 ± 0.0014	64,174,354	1	**<0.0001**
	Sex	−0.8748 ± 0.0009	850,181	1	**<0.0001**
	Urbanization level × Sex	−23.1300 ± 0.0009	633,904,345	1	**<0.0001**
Motion time	Urbanization level	−14.1700 ± 0.0089	2,539,792	1	**<0.0001**
	Sex	−18.9800 ± 0.0055	12,037,699	1	**<0.0001**
	Urbanization level × Sex	−68.6400 ± 0.0057	144,057,946	1	**<0.0001**
Inner square visit	Urbanization level	0.0297 ± 0.2643	0.0126	1	0.9105
	Sex	0.2687 ± 0.1928	1.9430	1	0.1633
	Urbanization level × Sex	−0.0485 ± 0.3358	0.0208	1	0.8852
Wall time	Urbanization level	−0.2437 ± 1.1432	0.0611	1	0.8048
	Sex	0.3578 ± 0.5456	0.4832	1	0.4870
	Urbanization level × Sex	−0.1436 ± 1.3654	0.0135	1	0.9075
Flight duration	Urbanization level	0.3503 ± 0.8129	0.1857	1	0.6666
	Sex	−0.6966 ± 0.5830	1.4279	1	0.2321
	Urbanization level × Sex	−1.0001 ± 0.5537	3.8542	1	**0.0496**
Flight distance	Urbanization level	−0.3528 ± 0.3224	1.1975	1	0.2738
	Sex	0.1883 ± 0.2048	0.8453	1	0.3579
	Urbanization level × Sex	−1.1994 ± 0.4176	4.2165	1	**0.0400**

## Data Availability

The data presented in this study are available on request from the corresponding author.

## Supplementary Materials

The following are available online at https://www.mdpi.com/article/10.3390/insects12070646/s1, Table S1: Spearman correlations between the tested behavioral measures (average of the two trials for each measures) within each personality dimension found in the *Carabus convexus* individuals sampled in rural and urban habitats. Values in bold denote significant (*p* < 0.05) correlations.
