# Supplementary material for: Are There Personality Differences between Rural vs. Urban-Living Individuals of a Specialist Ground Beetle, Carabus convexus?
Source: Insects. 2021 Jul 15;12(7):646. doi: 10.3390/insects12070646 (PMC8304727; doi:10.3390/insects12070646)
Supplement: Supplementary file 1 [file insects-12-00646-s001.zip › insects-1287673-supplementary.pdf]

Supplementary material to the article

Are there personality differences between rural vs. urban-living individuals of a specialist ground beetle, *Carabus convexus*?

by Tibor Magura, Szabolcs Mizser, Roland Horváth, Dávid D. Nagy, Mária Tóth, Réka Csicsek & Gábor L. Lövei

Insects

Table S1. Spearman correlations between the tested behavioural measures (average of the two trials for each measure) within each personality dimension found in *Carabus convexus* individuals collected in rural and urban habitats. Values in bold denote significant (p<0.05) correlations.

	Square visit	Covered distance	Motion time	Inner square visit	Wall time	Flight duration	Flight distance
Square visit	1.0000	**0.9963**	**0.9708**	**0.6822**	**-0.4458**	0.0657	0.2470
Covered distance	**0.9963**	1.0000	**0.9808**	**0.6595**	**-0.4539**	0.0823	0.2580
Motion time	**0.9708**	**0.9808**	1.0000	**0.6124**	**-0.4385**	0.1376	0.3008
Inner square visit	**0.6822**	**0.6595**	**0.6124**	1.0000	**-0.3856**	-0.0030	0.2034
Wall time	**-0.4458**	**-0.4539**	**-0.4385**	**-0.3856**	1.0000	-0.0740	-0.0134
Flight duration	0.0657	0.0823	0.1376	-0.0030	-0.0740	1.0000	**0.8552**
Flight distance	0.2470	0.2580	0.3008	0.2034	-0.0134	**0.8552**	1.0000
